# Pharmacological targeting of the GABA_B_ receptor alters *Drosophila's* behavioural responses to alcohol

**DOI:** 10.1111/adb.12725

**Published:** 2019-02-13

**Authors:** Daniel C. Ranson, Samir S. Ayoub, Olivia Corcoran, Stefano O. Casalotti

**Affiliations:** ^1^ Medicines Research Group University of East London London UK

**Keywords:** addiction behaviours, alcohol use disorder, CGP 54626, *Drosophila melanogaster*, GABA_B_ receptor, SKF 97541

## Abstract

When exposed to ethanol, *Drosophila melanogaster* display a variety of addiction‐like behaviours similar to those observed in mammals. Sensitivity to ethanol can be quantified by measuring the time at which 50% of the flies are sedated by ethanol exposure (ST50); an increase of ST50 following multiple ethanol exposures is widely interpreted as development of tolerance to ethanol. Sensitivity and tolerance to ethanol were measured after administration of the gamma‐aminobutyric acid receptor B (GABA_B_) agonist (SKF 97541) and antagonist (CGP 54626), when compared with flies treated with ethanol alone. Dose‐dependent increases and decreases in sensitivity to ethanol were observed for both the agonist and antagonist respectively. Tolerance was recorded in the presence of GABA_B_ drugs, but the rate of tolerance development was increased by SKF 97451 and unaltered in presence of CGP 54626. This indicates that the GABA_B_ receptor contributes to both the sensitivity to ethanol and mechanisms by which tolerance develops. The data also reinforce the usefulness of *Drosophila* as a model for identifying the molecular components of addictive behaviours and for testing drugs that could potentially be used for the treatment of alcohol use disorder (AUD).

## Graphical abstract

*Drosophila melanogaster* is an established model for alcohol addiction. We measured alcohol tolerance by recording the change in time in which half of a group of flies is sedated by alcohol vapours (change in ST50). We demonstrate that gamma‐aminobutyric acid receptor B (GABA_B_) agonist SKF 97541 and antagonist CGP 54626 decreased and increased ethanol sensitivity, respectively. Tolerance still developed in the presence and absence of the GABA_B_ ligands, but its rate was altered. This indicates that GABA_B_ receptors could potentially be a target for pharmacological intervention in alcohol addiction.

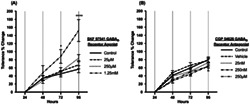

## 1 INTRODUCTION

Repeated alcohol consumption in humans leads to an increase in tolerance to alcohol, a known alcohol‐associated behaviour that can be replicated in animal models.^1^ Amongst the many pathways and genes that have been identified in animal models as being involved in initiating a change in alcohol‐associated behaviours, there has been no single molecular pathway or gene that has been shown to be solely responsible for reproducing or increasing tolerance and sensitivity to ethanol.^2^

In recent years, the ligand‐gated ion channel gamma‐aminobutyric acid receptor A (GABA_A_) has been a potential target for investigating the mechanisms of alcohol use disorder (AUD).^3^, ^4^, ^5^, ^6^, ^7^ It is also speculated that the GABA_A_ receptor is responsible for acute alcohol related changes in behaviour, but it is agreed amongst researchers that more work needs to be done before conclusive statements can be made.^8^ Less researched in the field of alcohol addiction when compared with the GABA_A_ receptor, is the gamma‐aminobutyric acid receptor B (GABA_B_); a class C metabotropic G–protein‐coupled receptor that has been evidenced to have an interesting and promising role in altering the sensitivity and preference to alcohol when pharmacologically targeted.^9^, ^10^, ^11^, ^12^, ^13^

The GABA_B_ receptor has been investigated and pharmacologically challenged with different GABA_B_ receptor ligands in different animal models for its potential role in modulating alcohol‐associated behaviours in AUD. Zaleski et al.,^14^ Cousins et al.,^9^ Dzitoyeva et al.,^10^ and Hwa et al.,^11^ have all reported on the effects of administering a GABA_B_ receptor ligand to overcome the effects of alcohol addiction‐like behaviours in both mammalian and invertebrate models. Likewise, the pharmacological activation of the GABA_B_ receptor has been shown to reduce the behavioural effects of addiction to both cocaine and nicotine.^9^, ^15^, ^16^ While these previous studies support the notion that GABA_B_ receptors play a role in addiction pathways, further investigations using GABA_B_ receptor drugs have the potential to elucidate the specific addiction mechanisms that are affected by GABA_B_ receptor activation or inhibition.

Among the wide variety of animal models employed in addiction research, *Drosophila melanogaster* has been identified as being a strong model for studying the acute and chronic effects of alcohol abuse.^17^, ^18^, ^19^ The underlying fundamental when using any animal model to replicate a human disease is to ensure simplicity, reproducibility, and mechanistic validity with respect to human behaviours, and *Drosophila*, in this instance, are a highly valuable model when used to study alcohol addiction‐like behaviours^18^, ^19^.

In this work, GABA_B_ receptor agonist, SKF 97541, and GABA_B_ receptor antagonist, CGP 54626, (previously used in *Drosophila* by Dzitoyeva et al., Dacks et al., and Root et al.,^10^, ^21^, ^22^) were used to target the GABA_B_ receptor to demonstrate that this receptor plays a role in alcohol sensitivity and tolerance. This novel research supports the possibility of targeting the GABA_B_ receptor as a strategy for the treatment of AUD.

## 2 MATERIALS AND METHODS

### 2.1 *Drosophila* husbandry

All flies used were Canton S wild‐type male *Drosophila* (#64349) obtained from Bloomington Drosophila Stock Centre, Indiana, USA. Flies were kept at 25°C with an average humidity of 60% to 80% on standard ready mixed dried food (Phillip Harris, UK). All flies tested were aged 1 to 5‐day old (1‐day old on first day of testing) and were collected the morning before testing via light CO_2_ anaesthesia to allow sufficient recovery after sedation.

### 2.2 Tolerance assay

The tolerance assay was conducted by following the methodology described by Maples and Rothenfluh.^23^ Male flies were sorted into groups of eight and were placed into vials containing standard food 24 hours prior to testing. This was completed so that the group of test flies could be transferred to the tolerance chamber with no CO_2_ anaesthesia. Five hundred microliters of 100% ethanol were added to the centre of a cotton vial plug, which was used to plug the tolerance chamber with the ethanol soaked side facing inwards. Flies were left to be exposed to the ethanol vapour until 50% of the total number of flies were sedated; this time point was defined as ST50 (time taken for 50% of flies in the vial to become sedated). Vials were tapped three times onto the lab bench once per minute to disorientate and startle the flies and to knock them to the bottom of the vial. Each vial was observed for 10 seconds, and the number of stationary flies was recorded every minute. The term “sedation” was determined by making clear distinguishable rules to follow; after flies had been knocked to the bottom of the vial, any fly not able to upright themselves within a 10 second observation time was determined as being sedated. Any leg movement or vibrating of the wings was ignored, and if the flies were still upon their backs after being startled to the bottom of the vial with the inability to self‐right, the fly was also recorded as being sedated.

Each assay was conducted at room temperature (with a control sample of eight 1‐ to 5‐day‐old male flies every time to ensure no bias was created with a change in room temperature) until the ST50 for each vial was achieved. Once sedated, flies were placed into an ethanol vapour free vial to recover at room temperature. After the flies had recovered and normal motility had resumed, they were housed back upon standard dry mix food at 25°C, and 24 hours later, the assay was repeated.

Tolerance was measured over a period of 4 days, and the ST50 was recorded for each vial. Tolerance to ethanol is identified as being an increase in ST50 over consecutive days following repeated ethanol exposures. This results in flies having a decreased sensitivity to ethanol, consequently resulting in an increased ST50 over time when compared with the first ethanol exposure on day one.^23^, ^24^, ^25^, ^26^

Data from the tolerance assay were collected by testing one vial of each condition on eight consecutive weeks. Each vial contained eight 1 to 5‐day‐old male flies (1‐day old on first day of testing) per vial. Mean values are N = 8 ± standard error of the mean (SEM).

### 2.3 Drug selection and delivery

**Figure 1:**
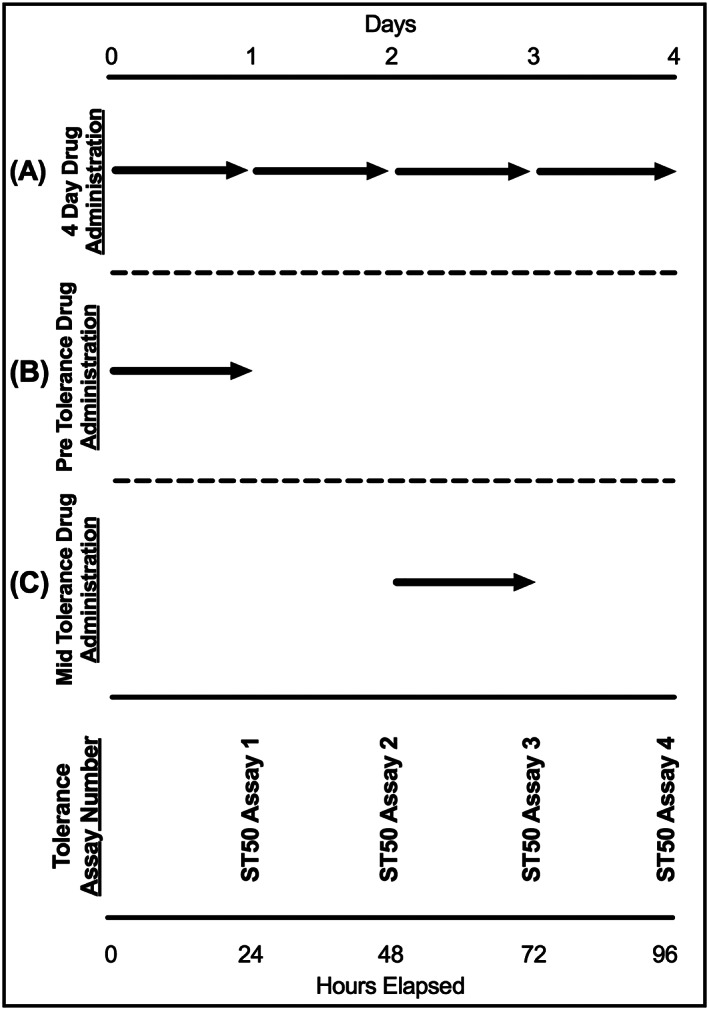
Administration schedule of ethanol and gamma‐aminobutyric acid receptor B (GABA_B_) drugs. All flies were exposed to ethanol vapour four times and their ST50 recorded (ST50 assay 1‐4). GABA_B_ drugs were administered to the fly via liquid food presented in capillary tubes to which the flies had access ad libitum. Three different GABA_B_ drug administration schedules were used: A, continuous administration: flies were fed with the drug for 24 hours before the first tolerance assay (when flies were naive to ethanol) and throughout the period in between consequent ST50 assays, B, preadministration: drugs were fed only for 24 hours before the first ST50 assay (when flies were naive to ethanol), C, mid administration: drugs were fed for 24 hours only between the second and third ST50 assay. Each arrow represents drug administration for a 24‐hour period

SKF 97541 is a potent and selective GABA_B_ receptor agonist, and CGP 54626 is a potent and selective GABA_B_ receptor antagonist (Tocris Biotechne, UK). SKF 97451 was dissolved in water at a stock concentration of 25 mM and CGP 54626 was dissolved in DMSO (Tocris Biotechne, UK) at a stock concentration of 25 mM. Flies were exposed to the drugs at the desired concentrations in the liquid food (5% yeast extract, 5% sucrose, 1% red coloured dye [Sigma Aldrich, UK]). Drug and control solutions were loaded into four 5 μL glass capillaries (Jaytec, UK) within a CAFÉ assay chamber as described by Ja et al.^27^ Flies were allowed to consume the liquid food via the four‐glass capillaries for 24‐hour *ad libitum* **.** Flies' abdomens were observed to see if they were red prior to testing; this was used as a positive indicator as to whether the flies had consumed/recently consumed the liquid food containing the red food colouring. The average consumption of liquid food was estimated from measuring the capillary meniscus over 24 hours, and it was estimated to be 0.42 μL ± 0.046 SEM with no significant difference in the consumption of different drug containing foods (Figure S1). Vials with flies that had less than four out of eight red abdomens were discarded. Control flies were fed the same liquid food with no added drug. A vehicle control group was treated with 0.002% DMSO (Tocris Biotechne, UK) which corresponds to the dose of DMSO delivered with CGP 54626. Liquid foods with/without the drug were given to *Drosophila* with different protocols: (a) for 24 hours prior and during the 4 days of tolerance testing, (b) only for 24 hours prior to tolerance testing, (c) only for 24 hours after the second ethanol exposure, prior to the day 3 ethanol exposure (Figure 1).

### 2.4 Locomotor activity assay

The locomotor climbing assay was constructed by wrapping 2‐cm graph paper with horizontal lines around the rear of a standard 9.5 × 2.5 cm vial (height × diameter). A vial was positioned in close proximity to a timer, and a camera was positioned to capture both the climbing assay vial and the timer within one frame. The camera was set to record once the flies were transferred within the assay vial and cameras field of view. All flies were then tapped to the bottom of the vial and at the time of doing so, the timer was manually started.

The frames of video were visualised through QuickTime Player Version 10.4 (Apple Inc, USA) by scanning through frame by frame until all flies had crossed the 8 cm mark on the vial or 15 seconds had elapsed, whichever was sooner. The time at which all flies had crossed the 8‐cm mark was then recorded.

### 2.5 Ethanol concentration measurement

Ethanol absorption was measured with a spectrophotometric assay as described previously by Moore et al.^28^ Thirty 1 to 5‐day‐old male flies were exposed to 500 μL of 100% ethanol in a tolerance chamber (as described above). After 30 minutes, flies were frozen in dry ice and were homogenised in 300 μL of ice‐cold Tris‐HCl (pH 7.5). The homogenate was centrifuged at 4500 revolutions per minute for 20 minutes at 4°C. Five microlitres of supernatant was added to 495 μL of ethanol assay reagent (Sigma, UK). The manufacturers protocol and calculation instructions were followed. To calculate the ethanol concentration in individual flies, the volume of one fly was estimated to be 2 μL.^28^ The estimated ethanol concentration in one individual fly after 30 minutes was calculated to be 6.5 mM. A control group (no ethanol vapour) was also assayed, and this result was negative (Figure S2).

### 2.6 Statistical analysis

All graphs and analysis were made and executed using the Prism for Mac OS X Version 7.0 (GraphPad Software Inc, USA).

The change in sensitivity (in minutes) was calculated by subtracting the ST50 value of the treated flies by the ST50 value for the control flies of the same day of treatment. The percentage change in sensitivity from day 2 to day 4 (tolerance) was calculated by the formula ([(‘Day X' ST50/Day 1 ST50] −1) × 100. ANOVA analysis was carried out to determine statistical significance.

## 3 RESULTS

### 3.1 Development of tolerance to ethanol

**Figure 2:**
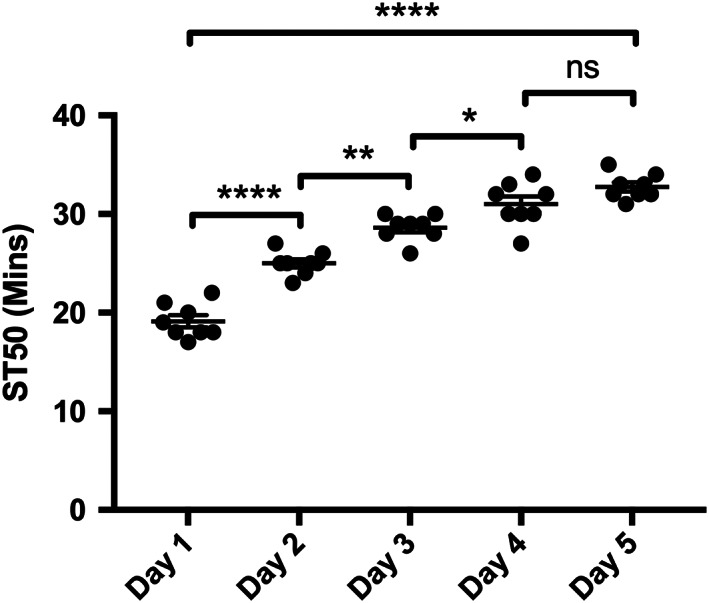
Tolerance development in wild‐type *Drosophila* **.** The time at which half of each group of flies were sedated (ST50) when exposed to 500 μL of 100% EtOH was recorded for five consecutive days. Flies were naive to ethanol prior to day 1. Values are mean ± SEM with N = 8 (eight vials with eight flies per vial). 1‐way ANOVA with Tukey vs previous day. Statistical comparison indicated by lines. * = <0.05, ** = <0.01, **** = <0.0001

Repeated exposures to ethanol in wild type flies leads to a reduced sensitivity to its sedative effect. Tolerance development is defined as a change in the time (ST50) needed for half of a set of flies exposed to ethanol vapour to become sedated as compared with the first ethanol exposure. ST50 was measured in sets of eight male flies and was shown to increase over a period of 5 days with daily ethanol exposures (Figure 2). These findings are consistent with previous reports confirming that *D*. *melanogaster* are a suitable model to study the development of alcohol tolerance as an addiction like behaviour, similar to that seen in humans.^20^, ^23^, ^24^, ^26^, ^29^

### 3.2 Tolerance development with continuous administration of agonist SKF 97541

**Figure 3:**
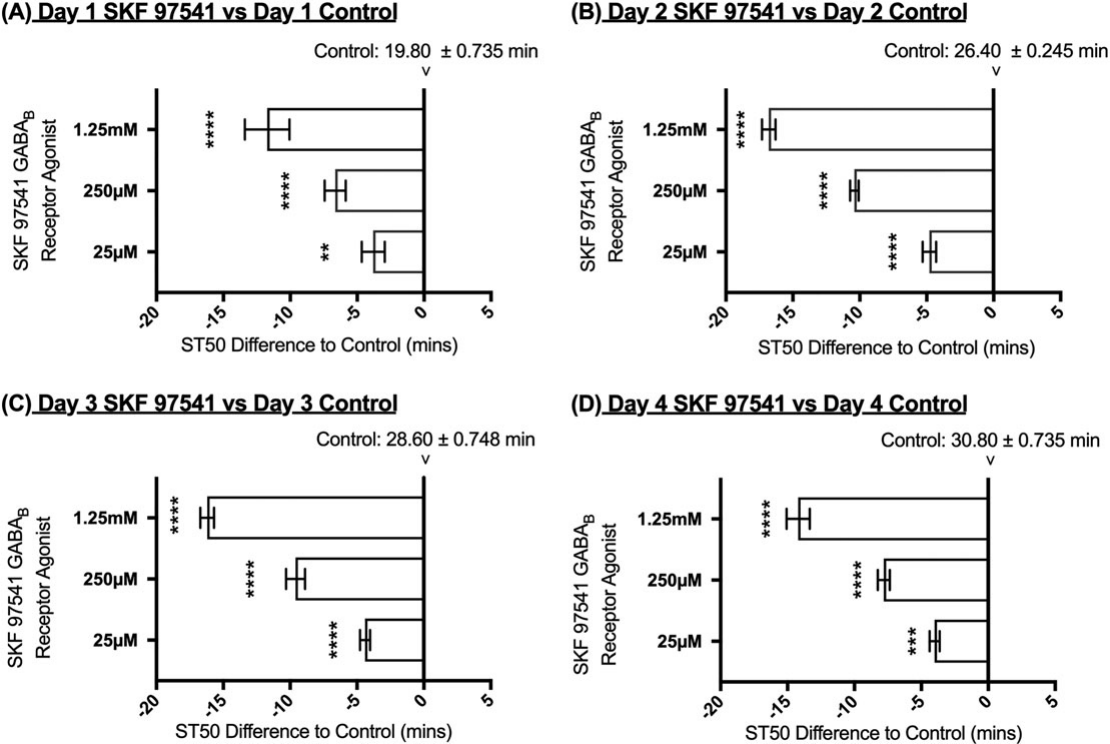
Effect of continuous administration of SKF 97451 on ST50. Flies were fed SKF 97451 for 24 hours before the first ethanol exposure and for the whole period in between the four ethanol exposures. Horizontal bars represent changes in ST50 in minutes in flies fed different concentration of SKF 97451 when compared with the control of the same day. N = 8 (eight vials with eight flies per vial). The ST50 of the control flies is indicated in minutes mean ± SEM at the zero‐difference point for each consecutive day. Averages ± SEM. 2‐way ANOVA with Tukey vs control of that day. ** = <0.01, *** = <0.001, **** = <0.0001

The GABA_B_ receptor agonist SKF 97541 was dissolved in liquid food and was administered via glass capillaries *ad libitum* for 24 hours prior to the first ST50 assay and during the subsequent 3 days of testing (Figure 1A for drug administration schedule). SKF 97541 agonist was effective at reducing ST50 and thus increasing the sensitivity of the flies to ethanol (Figure 3). On each day of testing, at all drug concentrations used, ST50 values expressed in minutes were lower in the drug treated flies than in untreated control flies by comparing values for the same day of exposure.

**Figure 4:**
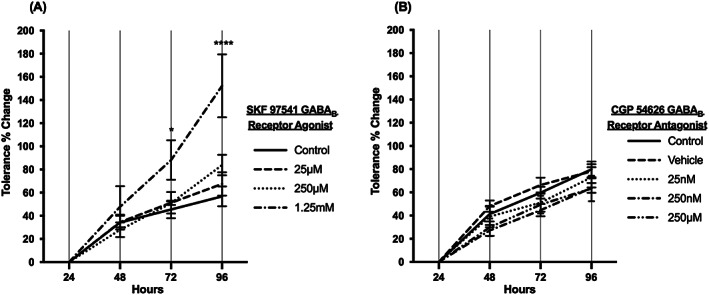
Change in tolerance after continuous administration of SKF 97541 and CGP 54626**.** Flies were fed either with A, SKF 97541 or B, CGP 54626 for 24 hours before the first ethanol exposure and for the whole period in between the four exposures. N = 8 (eight vials with eight flies per vial). Averages ± SEM. 2‐way ANOVA. Significance reported is SKF 97541 1.25 mM vs water control * = <0.05, **** = <0.0001. The percentage change in sensitivity (tolerance) was calculated by the formula ([‘Day X' ST50/Day 1 ST50] − 1) × 100

It can be observed that whilst SKF 97541 significantly reduces the time in which sedation occurs by increasing the sensitivity of the flies to the ethanol vapour, the flies do retain the ability to develop tolerance to ethanol as demonstrated by the ST50 percentage change measured at each of the test days when compared with the first day of exposure (Figure 4A). It should also be noted that tolerance development was increased by SKF 97541 by the end of the 4‐day exposure. The tolerance percentage change increased from 56.72% (water control) on day 4, to 67.53%, 83.81%, 152.31% (SKF 97541: 25 μM, 250 μM, 1.25 mM). The 1.25 mM concentration induced change that was statistically significant on day 3 and 4 (*p* <0.05, *p* <0.001 respectively).

### 3.3 Tolerance development with continuous administration of antagonist CGP 54626

**Figure 5:**
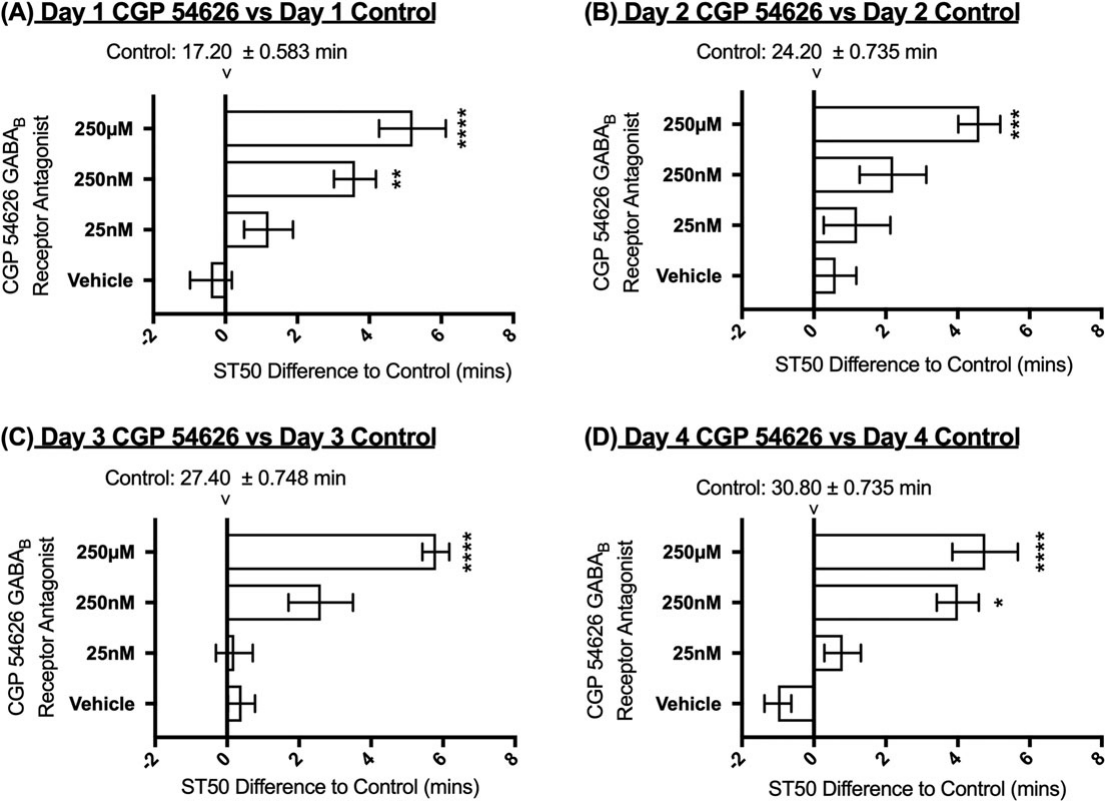
Effect of continuous administration of CGP 54626 on ST50**.** Flies were fed with CGP 54626 for 24 hours before the first ethanol exposure and for the whole period in between the four ethanol exposures. Horizontal bars represent changes in ST50 in minutes in flies fed different concentration of CGP 54626 when compared with the control of the same day. N = 8 (eight vials with eight flies per vial). The ST50 of the control flies is indicated in minutes ± SEM at the zero difference point for each consecutive day. Averages ± SEM. 2‐way ANOVA with Tukey vs. control of that day. * = <0.05, ** = <0.01, *** = <0.001, **** = <0.0001

To further explore the role of the GABA_B_ receptor in response to ethanol exposure, the GABA_B_ antagonist CGP 54626 was administered to the flies with the same protocol used for the agonist (Figure 1A, Figure 4B and Figure 5).

Administration of CGP 54626 for 24 hours prior to day 1 of the ST50 assay and continually administered for the following 3 days of testing resulted in a significant decrease in sensitivity (increase in ST50) at concentrations of 250 nM and 250 μM (Figure 5). The relative increase in ST50 (tolerance development) occurred in the presence of the drug despite the already higher ST50 values. Thus, tolerance development can occur in the presence of the agonist and antagonist despite the fact that each drug reduced and increased ST50 values respectively. However, unlike for SKF 97541 there was no significant effect of CGP 54626 on the rate of tolerance development (Figure 4).

### 3.4 SKF 97541 agonist administration prior to and during tolerance development

**Figure 6:**
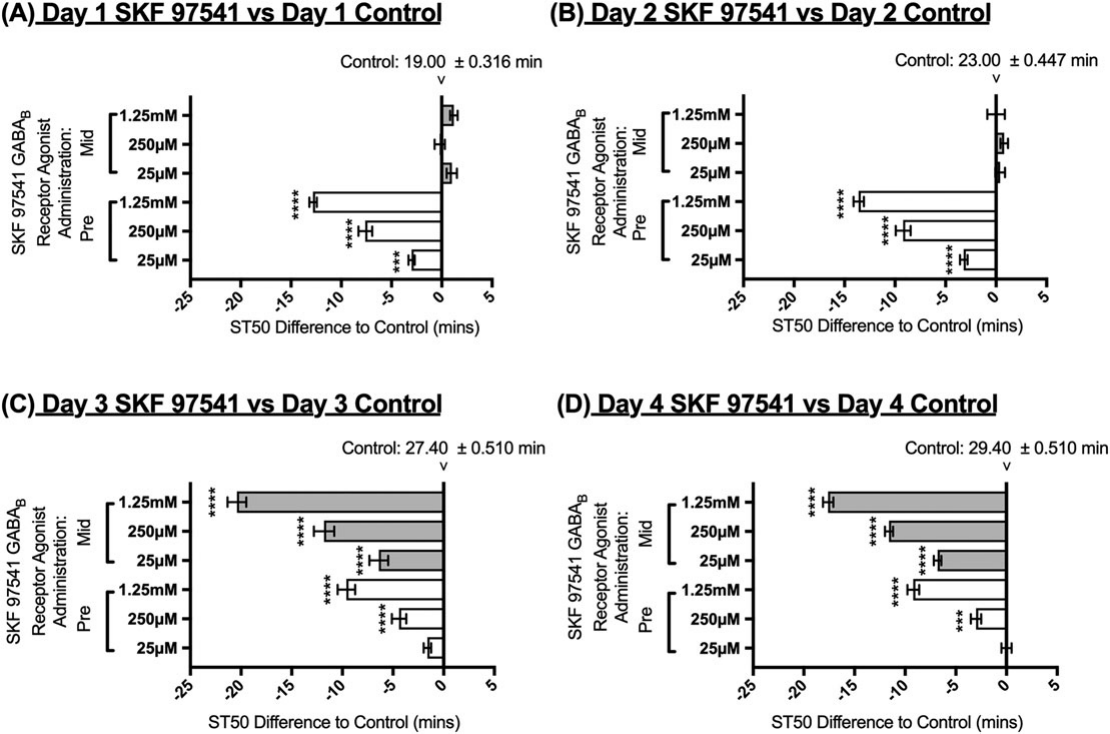
Effect of SKF 97541 pre‐administration and mid‐administration on sedation time (ST50). Flies were fed with SKF 97451 only for 24 hours prior to the 4‐day tolerance assay (pre‐administration, white bars) or for 24 hours in between the second and third day of the 4‐day tolerance assay (mid‐administration, shaded bars). Horizontal bars represent changes in ST50 in minutes in SKF 97451‐treated flies as compared with control untreated flies for the same day of the tolerance assay. N = 8 (eight vials with eight flies per vial). The ST50 of the control flies is indicated in minutes ± SEM at the zero‐difference point for each consecutive day. Averages ± SEM. 2‐way ANOVA with Tukey vs control of that day. *** = <0.001, **** = <0.0001

In order to investigate the continuity and stability on the effect of the agonist SKF 97541, the drug was administered for just 24 hours prior to the first tolerance assay (Figure 1B for administration schedule and Figure 6).

SKF 97451 administered at concentrations of 25 μM, 250 μM, and 1.25 mM before alcohol exposures significantly reduced ST50 when measured 24 and 48 hours after drug administration when compared with untreated flies for the same day of ethanol exposure (Figure 6A,B). On day 3 and 4, 72 and 96 hours after the initial drug administration, only the 250 μM and 1.25 mM doses of agonist remained effective at significantly reducing ST50 when compared with the water control for that day (Figure 6C,D). Comparison of Figure 6A and D indicates that the efficacy of the drug, when administered prior to day 1, decreases over days in a dose‐dependent manner. The ST50 percentage differences (not shown in Figure 6) between day 1 and day 4 are 54.2%, 43.2%, and 30.7% for control, 25 μM, 250 μM, and 1.25 mM respectively. The data therefore indicates that the effect of SKF 97451 is dependent on the drug being present at the time of the tolerance assay, and although we have not measured drug kinetics, it appears that its effect diminishes over time.

In order to further investigate whether SKF 97451 plays an active role in tolerance behaviour development, flies were allowed to develop tolerance to ethanol with two ethanol exposures over 48 hours, followed by 24 hours of *ad libitum* administration of SKF 97451. Flies were finally tested for tolerance over the next 48 hours after drug administration (Figure 1C for administration schedule and Figure 6). SKF 97451 concentrations of 25 μM, 250 μM, and 1.25 mM were able to significantly reduce the time to sedation values when administered after two alcohol exposures (Figure 6C,D). This highlights that the SKF 97541 agonist is effective at increasing the flies sensitivity to ethanol both prior to the initial tolerance development, and during the tolerance development when the flies sensitivity has already been modified.

### 3.5 CGP 54626 antagonist administration prior to and during tolerance development

**Figure 7:**
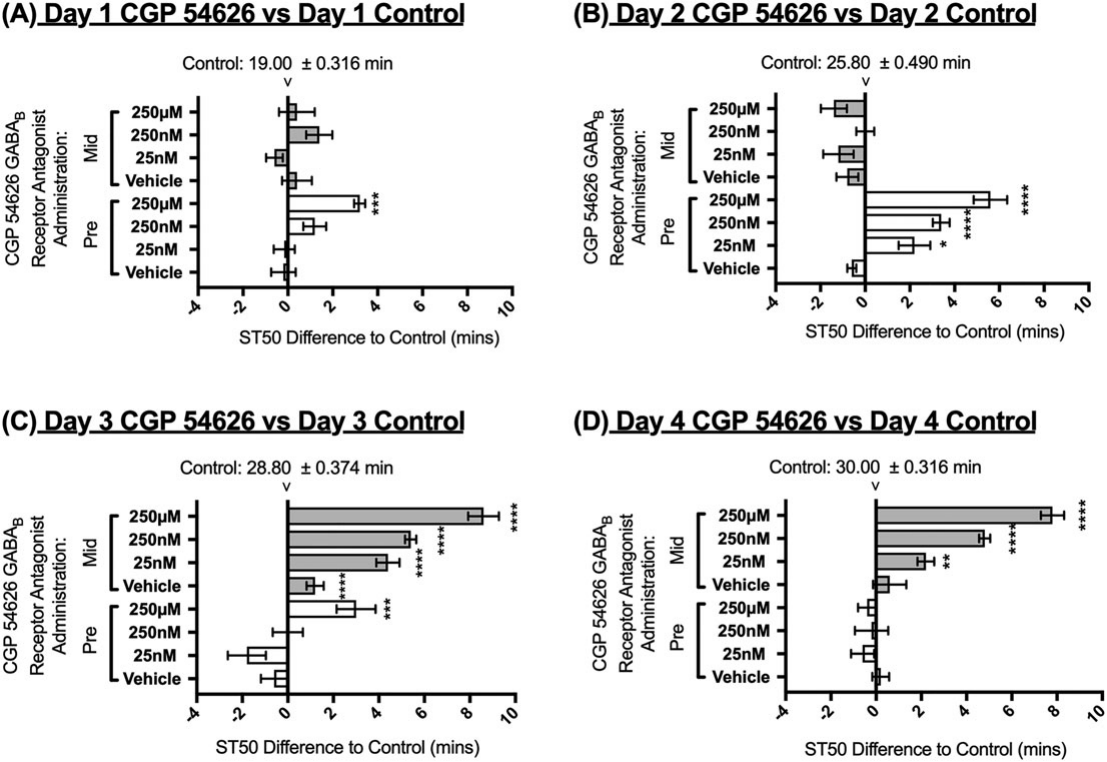
Effect of CGP 54626 pre‐administration and mid‐administration on sedation time (ST50). Flies were fed with CGP 54626 only for 24 hours prior to the 4‐day tolerance assay (pre‐administration, white bars) or for 24 hours in between the second and third day of the 4‐day tolerance assay (mid‐administration, shaded bars). Horizontal bars represent changes in ST50 in minutes in CGP 54626‐treated flies as compared with control‐untreated flies for the same day of the tolerance assay. N = 8 (eight vials with eight flies per vial). The ST50 of the control flies is indicated in minutes ± SEM at the zero‐difference point for each consecutive day. Averages ± SEM. 2‐way ANOVA with Tukey vs control of that day. * = <0.05, ** = <0.01, *** = <0.001, **** = <0.0001

Similarly to the experiments described above for SKF 97451, the GABA_B_ antagonist CGP 54626 was administered to the flies for 24 hours only prior to the four consecutive days of tolerance testing or for 24 hours only after the second day of tolerance testing (Figure 1B‐C for administration schedule). The 24‐hour administration of CGP 54626 prior to tolerance testing was effective at increasing the flies ST50 at a concentration of 250 μM on the first day of testing (Figure 7A). Notably, on the second day of the tolerance assay, all three concentrations tested (25 nM, 250 nM, and 250 μM) were significantly effective at decreasing the sensitivity to ethanol, thus increasing ST50 values when compared with the control for that respective day (Figure 7B). The effect of the drug was diminished to zero over the next 2 days of testing, and this indicates that the drug takes more than 24 hours to reach full efficacy before starting to lose its effect (Figure 7C‐D).

When CGP 54626 was administered after 2 days of ethanol tolerance, the ST50 values were increased to highly significant values when compared with the control for day 3 at all the CGP 54246 concentrations used, before slowly losing efficacy on day 4 (2 days/48 hours after initial administration) and there being an evident decrease in significance when compared with the control for day 4 in a dose dependent manner (Figure 7C,D).

## 4 DISCUSSION

Within this study, we have used a tolerance assay as described by Maples and Rothenfluh^23^ to show that *D*. *melanogaster* can develop tolerance with repeated exposures over consecutive days, mimicking an alcohol‐related behaviour that is seen with AUDs in humans (Figure 2). The *Drosophila* model allows pharmacological interventions that can aid in dissecting the mechanisms of tolerance development.^23^, ^24^, ^26^ In this work, we have used GABA_B_ receptor ligands to explore tolerance development. The range of concentrations of drug used were determined by toxicity studies where flies survived for more than 96 hours at the doses used (data not shown) and by negative geotaxis assays to determine that the drugs did not cause substantial effect on locomotor activity (Figure S3). The concentration of 1.25 mM SKF 97451 did affect locomotor activity; however, the results were retained in this study because it was of interest to determine what effect this higher concentration had when the files were exposed only for 24 hours pre or mid tolerance development (Figure 6). Additionally, the fact that tolerance developed even after continuous administration of this high concentration (Figure 3) indicates that the effect on locomotor activity and ST50 measurement is not necessarily correlated.

Whilst the idea that the GABA_B_ receptor could be used as a potential drug target to combat alcohol addiction like behaviours is not novel,^9^, ^10^, ^11^, ^16^, ^21^ this study provides direct evidence of an agonist and an antagonist ligand that are capable of increasing and decreasing sensitivity to ethanol vapour respectively after different numbers of alcohol exposures (Figures 3, 5, 6, 7).

The data presented indicate that the GABA_B_ drugs affect the sensitivity to alcohol by reducing/increasing the time to sedation, while permitting tolerance development to occur (Figure 4A,B). It should also be noted that the agonist SKF 97451 decreased the ST50 and altered the rate at which tolerance (defined as an increase in ST50) developed after four ethanol sensitivity assays. This novel data reinforce the concept that the development of tolerance is a multifactorial process where GABA_B_ receptors (like other receptor systems) contribute to, but do not fully determine the development of tolerance. Whilst it is known that ethanol increases GABA release^30^ and that the function of GABA_A_ receptors is affected by ethanol,^3^, ^8^ it is important to also fully characterise the role of GABA_B_ receptors in ethanol‐induced processes. The novel contribution of this work is that the modulation of the GABA_B_ receptors alters the sensitivity to ethanol as well as affecting the rate of tolerance development. There are potentially different mechanisms by which modulation of the GABA_B_ receptors could affect the change in tolerance development, which include modulation of GABA or other neurotransmitter release in the brain, and alcohol metabolism within the brain and other organs.

Prior to the successful cloning of the GABA_B_ receptor subunits by Mezler, Müller and Raming,^31^ *Drosophila* was thought to lack GABA_B_ receptors, as the GABA_B_ receptor agonist Baclofen has been reported to have no effect in *Drosophila*.^13^ However, the two pharmacological probes (agonist SKF 97541 and antagonist CGP 54626) used here have been reported to work as a specific GABA_B_ receptor agonist and antagonist in *Drosophila*.^10^, ^21^, ^22^ It still remains to be established however, as to why baclofen does not appear to be functional in *Drosophila*.

The ultimate goal of alcohol addiction research is to resolve addiction‐like behaviours in individuals with AUD. This work has strengthened the possibility that the GABA_B_ receptor could be pharmacologically targeted to improve AUD related behaviour. However, further investigations on the role of GABA_B_ receptors are required. The *Drosophila* model and the vast genetic tools that have been made available by the *Drosophila* research community have the potential to identify pharmacological strategies that could then be tested and applied in human subjects.

## AUTHOR CONTRIBUTIONS

DCR and SOC were responsible for the study concept and design. DCR was responsible for the acquisition of *Drosophila* tolerance and locomotor activity data. DCR, SSA, OC, and SOC assisted with the interpretation of the findings, and DCR created the figures and completed the statistical analysis. DCR and SOC drafted the manuscript. SSA and OC provided critical revisions of the manuscript. All authors critically reviewed content and approved the final version for publication.

## Supporting information

Figure S1: Consumption of liquid foods was measured for 24 h by measuring the capillary meniscus before and after the 24 h period in which flies were housed in the feeding vial. Values are mean ± SEM with *n* = 5 (5 vials with 8 flies per vial). 1‐way ANOVA with Bonferroni multiple comparison shows no significant difference overall, indicated by horizontal line, (*p* = 0.1989) or between any two conditions (*p* > 0.05). The 30 vials (5x 6 conditions shown) were averaged to calculate consumption per fly (0.42 μl of food in 24 h per fly).
Figure S2: Ethanol concentration per fly after 30 minutes of sensitivity ST50 assay when exposed to 500 μL of 100% EtOH or distilled water. Flies were naïve to ethanol prior to this assay. Values are mean ± SEM with *n* = 2 (8 vials with 8 flies per vial). 1‐way ANOVA. * = <0.05.
Figure S3: Time taken for flies to climb 8 cm in a 10 cm high vial after 24 hour administration of **(a)** SKF 97541 GABA_B_ receptor agonist or **(b)** CGP 54626 GABA_B_ receptor antagonist. *n* = 2 (2 vials with 8 flies per vial). Triplicate readings were taken for each vial and the average recorded with SEM. 1‐way ANOVA with Dunnetts vs control of that day with no significance recorded. Flies were given 15 seconds to move 8 cm horizontally before the timer was stopped. Agonist administered 1.25mM flies did not move past 2 cm for 3 days within the allotted 15 second period.
