# Supplementary figures and images for: Pharmacological targeting of the GABAB receptor alters Drosophila's behavioural responses to alcohol
Source: Addict Biol. 2019 Feb 13;25(2):e12725. doi: 10.1111/adb.12725 (PMC7050513; doi:10.1111/adb.12725)

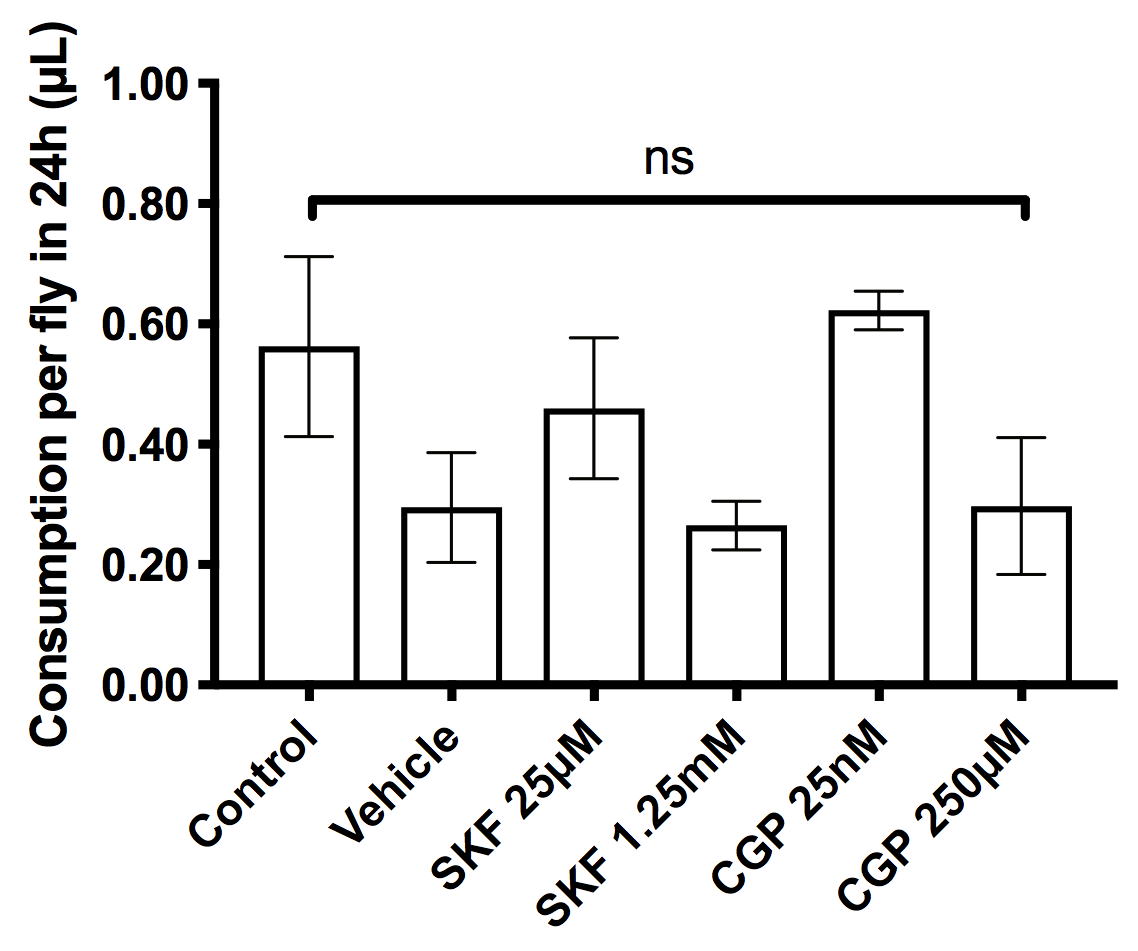

Supplement: Supplementary file 1 — Figure S1: Consumption of liquid foods was measured for 24 h by measuring the capillary meniscus before and after the 24 h period in which flies were housed in the feeding vial. Values are mean ± SEM with n = 5 (5 vials with 8 flies per vial). 1‐way ANOVA with Bonferroni multiple comparison shows no significant difference overall, indicated by horizontal line, (p = 0.1989) or between any two conditions (p > 0.05). The 30 vials (5x 6 conditions shown) were averaged to calculate consumption per fly (0.42 μl of food in 24 h per fly). Figure S2: Ethanol concentration per fly after 30 minutes of sensitivity ST50 assay when exposed to 500 μL of 100% EtOH or distilled water. Flies were naïve to ethanol prior to this assay. Values are mean ± SEM with n = 2 (8 vials with 8 flies per vial). 1‐way ANOVA. * = <0.05. Figure S3: Time taken for flies to climb 8 cm in a 10 cm high vial after 24 hour administration of (a) SKF 97541 GABAB receptor agonist or (b) CGP 54626 GABAB receptor antagonist. n = 2 (2 vials with 8 flies per vial). Triplicate readings were taken for each vial and the average recorded with SEM. 1‐way ANOVA with Dunnetts vs control of that day with no significance recorded. Flies were given 15 seconds to move 8 cm horizontally before the timer was stopped. Agonist administered 1.25mM flies did not move past 2 cm for 3 days within the allotted 15 second period. [file ADB-25-e12725-s001.zip › ADB_12725-Supp_0002.tiff]

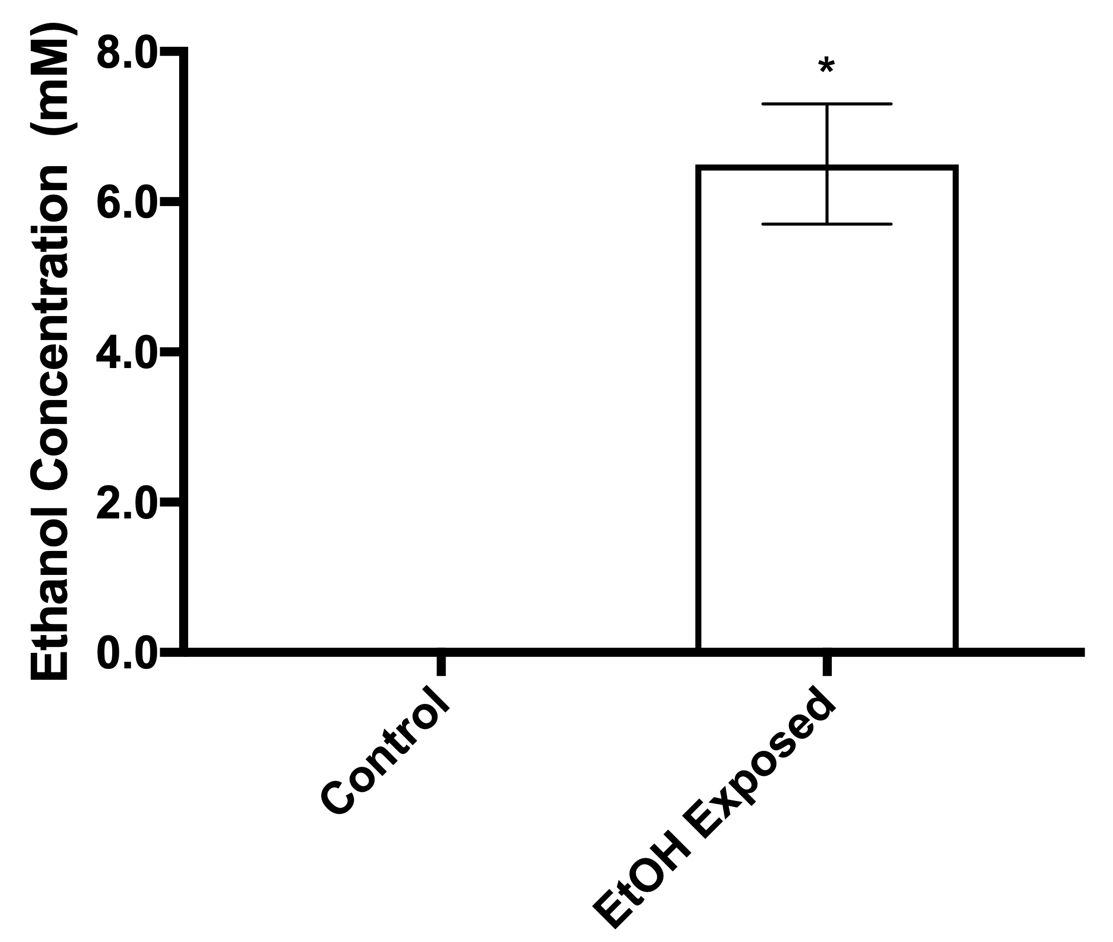

Supplement: Supplementary file 1 — Figure S1: Consumption of liquid foods was measured for 24 h by measuring the capillary meniscus before and after the 24 h period in which flies were housed in the feeding vial. Values are mean ± SEM with n = 5 (5 vials with 8 flies per vial). 1‐way ANOVA with Bonferroni multiple comparison shows no significant difference overall, indicated by horizontal line, (p = 0.1989) or between any two conditions (p > 0.05). The 30 vials (5x 6 conditions shown) were averaged to calculate consumption per fly (0.42 μl of food in 24 h per fly). Figure S2: Ethanol concentration per fly after 30 minutes of sensitivity ST50 assay when exposed to 500 μL of 100% EtOH or distilled water. Flies were naïve to ethanol prior to this assay. Values are mean ± SEM with n = 2 (8 vials with 8 flies per vial). 1‐way ANOVA. * = <0.05. Figure S3: Time taken for flies to climb 8 cm in a 10 cm high vial after 24 hour administration of (a) SKF 97541 GABAB receptor agonist or (b) CGP 54626 GABAB receptor antagonist. n = 2 (2 vials with 8 flies per vial). Triplicate readings were taken for each vial and the average recorded with SEM. 1‐way ANOVA with Dunnetts vs control of that day with no significance recorded. Flies were given 15 seconds to move 8 cm horizontally before the timer was stopped. Agonist administered 1.25mM flies did not move past 2 cm for 3 days within the allotted 15 second period. [file ADB-25-e12725-s001.zip › ADB_12725-Supp_0003.tiff]

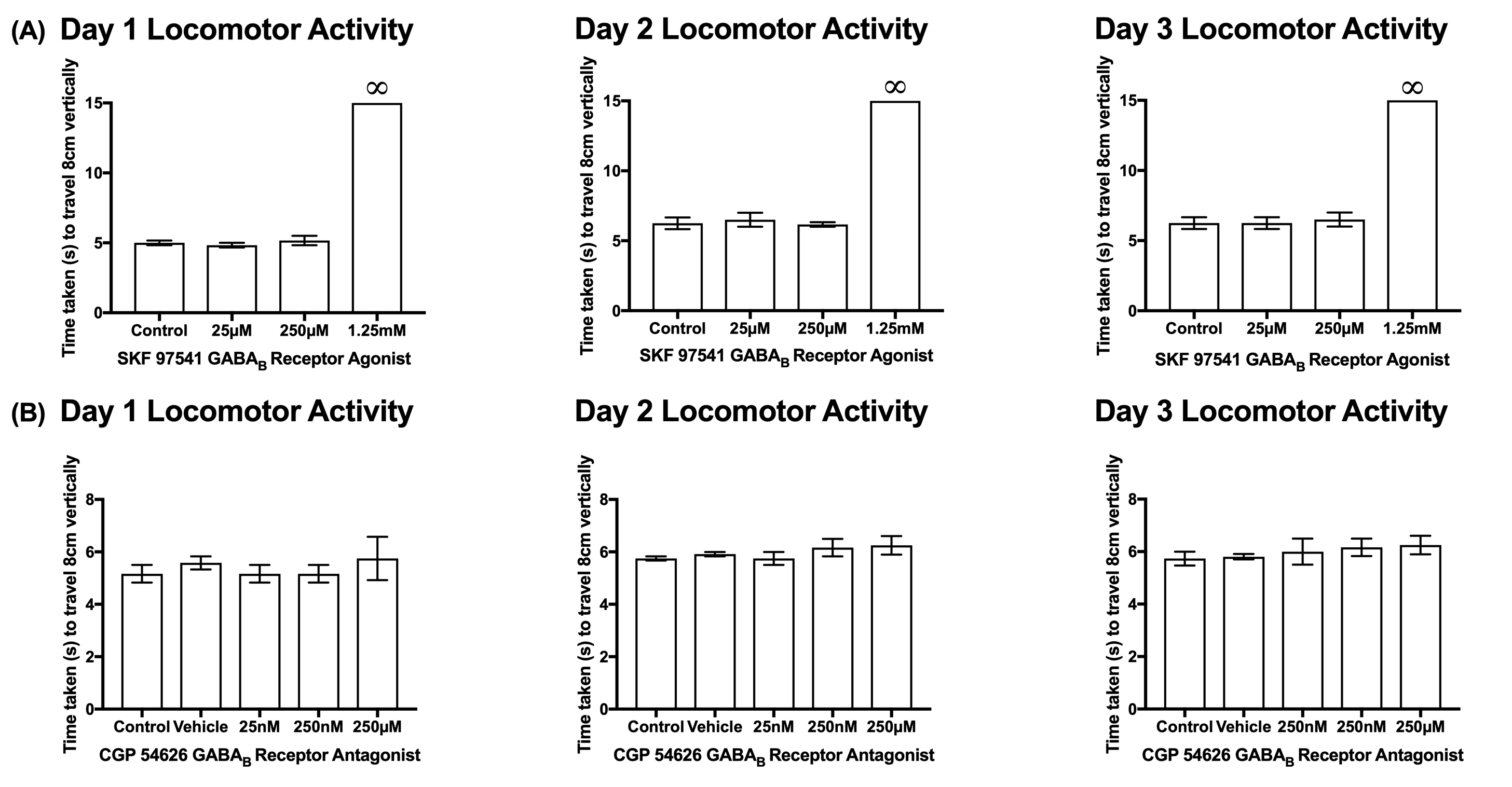

Supplement: Supplementary file 1 — Figure S1: Consumption of liquid foods was measured for 24 h by measuring the capillary meniscus before and after the 24 h period in which flies were housed in the feeding vial. Values are mean ± SEM with n = 5 (5 vials with 8 flies per vial). 1‐way ANOVA with Bonferroni multiple comparison shows no significant difference overall, indicated by horizontal line, (p = 0.1989) or between any two conditions (p > 0.05). The 30 vials (5x 6 conditions shown) were averaged to calculate consumption per fly (0.42 μl of food in 24 h per fly). Figure S2: Ethanol concentration per fly after 30 minutes of sensitivity ST50 assay when exposed to 500 μL of 100% EtOH or distilled water. Flies were naïve to ethanol prior to this assay. Values are mean ± SEM with n = 2 (8 vials with 8 flies per vial). 1‐way ANOVA. * = <0.05. Figure S3: Time taken for flies to climb 8 cm in a 10 cm high vial after 24 hour administration of (a) SKF 97541 GABAB receptor agonist or (b) CGP 54626 GABAB receptor antagonist. n = 2 (2 vials with 8 flies per vial). Triplicate readings were taken for each vial and the average recorded with SEM. 1‐way ANOVA with Dunnetts vs control of that day with no significance recorded. Flies were given 15 seconds to move 8 cm horizontally before the timer was stopped. Agonist administered 1.25mM flies did not move past 2 cm for 3 days within the allotted 15 second period. [file ADB-25-e12725-s001.zip › ADB_12725-Supp_0004.tiff]
